# Inhibition of canonical WNT/β-catenin signaling is involved in leflunomide (LEF)-mediated cytotoxic effects on renal carcinoma cells

**DOI:** 10.18632/oncotarget.10409

**Published:** 2016-07-06

**Authors:** Yicheng Chen, Qiaoli Huang, Hua Zhou, Yueping Wang, Xian Hu, Tao Li

**Affiliations:** ^1^ Department of Urology, Sir Run-Run Shaw Hospital, College of Medicine, Zhejiang University, Hangzhou, Zhejiang 310016, China; ^2^ Department of Biology, College of Chemistry and Life Sciences, Zhejiang Normal University, Jinhua, Zhejiang 321004, China; ^3^ Department of Urology, Wuyi First People's Hospital, Wuyi, Zhejiang 321200, China; ^4^ Department of Plastic Surgery, Sir Run-Run Shaw Hospital, College of Medicine, Zhejiang University, Hangzhou, Zhejiang 310016, China

**Keywords:** leflunomide, renal cell carcinoma, cytotoxicity, WNT/β-catenin signaling

## Abstract

Leflunomide (LEF), an inhibitor of dihydroorotate dehydrogenase (DHODH) in pyrimidine biosynthetic pathway, is an immunomodulatory agent approved for the treatment of rheumatoid arthritis. In this study, we show that LEF significantly reduced cell proliferation of renal carcinoma cells in a concentration-dependent manner. LEF at 50 μM induced S-phase arrest and autophagy. Higher doses of LEF (>50 μM) effectively induced cell apoptosis. Modulating the concentration of LEF resulted in distinct effects on the expression of regulatory proteins associated with cell cycle, apoptosis, and autophagy. In particular, high concentrations of LEF inhibited canonical WNT signaling by promoting nucleo-cytoplasmic shuttling and proteasome-dependent degradation of β-catenin. Mechanistic studies showed that the repression of AKT activation partly accounted for LEF-mediated WNT inhibition. Gene expression microarray revealed that LEF treatment greatly inhibited the expression of *FZD10* gene, a receptor mediating WNT/β-catenin activation. *In vivo* xenograft study in NOD/SCID mice further validated the inhibitory effects of LEF on tumor growth and Wnt/β-catenin signaling. However, LEF treatment also triggered cell autophagy and elevated the expression of *WNT3a*, which ameliorated its cytotoxic effects. The combination of LEF with a WNT inhibitor IWP-2 or autophagy inhibitor HCQ could yield an enhanced anti-tumor outcome. Taken together, these results identify the potential utility and pharmacological feature of LEF in the chemotherapy of renal cell carcinoma (RCC).

## INTRODUCTION

Renal cell carcinoma (RCC) is the most common neoplastic disease in the adult kidney, accounting for approximately 2-5% of adult malignancies. Metastasis may be present at the RCC diagnosis and such cases are resistant to radiotherapy and chemotherapy. Since 2005, mRCC treatment has focused on targeted therapies that inhibit vascular endothelial growth factor receptors, such as bevacizumab, sorafenib, sunitinib and pazopanib, or alternatively mTOR (mammalian target of rapamycin) kinase, such as temsirolimus and everolimus. However, the benefits of such treatments are limited. Therefore, there remains an unmet need for therapeutic drugs that might improve the metastasis renal cell carcinoma (mRCC) outcome [1].

Leflunomide (LEF) as a synthetic isoxazole-derivative drug is widely used in the prevention and treatment of autoimmune disorders and allograft rejection. LEF is rapidly absorbed and converted into the active metabolite termed teriflunomide or A771726 through gut wall nonenzymatic mechanisms and first-pass hepatic metabolism. The drug is eventually eliminated renally or excreted into the feces. A771726 is almost entirely bound to albumin, leading to a plasma half-life of 15.7 days. Steady-state plasma concentrations up to 200 μM after oral ingestion can be achieved and severe side effects are seldom observed [2, 3]. LEF and teriflunomide have been characterized as potent inhibitors of dihydroorotate dehydrogenase (DHODH), a rate-limiting enzyme in the synthesis pathway of pyrimidines. Upon stimulation with mitogens, dividing cells significantly expand their pyrimidine pool in a manner dependent on DHODH. Thereby, LEF can exert anti-proliferative roles by blocking pyrimidine synthesis in a non-competitive manner. Based on its reliable ability to antagonize the proliferation of mitogen-stimulated lymphocytes, the drug was first approved for the treatment of rheumatoid arthritis (RA) in 1998. Accumulating clinical evidence validate that LEF can effectively relieve symptoms and delay radiographic progression of rheumatoid arthritis without considerable side effects. Besides, LEF possesses other appealing clinical advantages, such as inexpensive cost, long half-life and weak interaction with the cytochrome system. Owing to its profitable properties, LEF attracted great interest in applications other than RA treatment in the last few years [3].

Indeed, LEF and its active metabolite have also been reported to show antitumor potential through repression of cancer cell proliferation and induction of apoptosis. Case series have demonstrated therapeutic benefits of LEF in the treatment of multiple myeloma, breast cancer, lymphocytic leukemia, neuroblastoma, erythroleukemia, hepatocellular carcinoma, prostate cancer, melanoma, medullary thyroid cancer, and so on [4-16]. However, whether LEF has any inhibitory effect on renal cell carcinoma is yet not clearly elucidated.

In this study, we demonstrate that modulating the concentration of LEF inhibits cell viability of renal carcinoma cells via inducing cell cycle arrest, autophagy, and apoptosis. Mechanistic studies showed that LEF at high concentrations effectively interrupted canonical WNT/β-catenin signaling. In particular, LEF treatment significantly altered the expression of *FZD10* and other members of WNT/β-catenin signaling.

## RESULTS

### LEF inhibits cell growth in RCC cell lines

**Figure 1:**
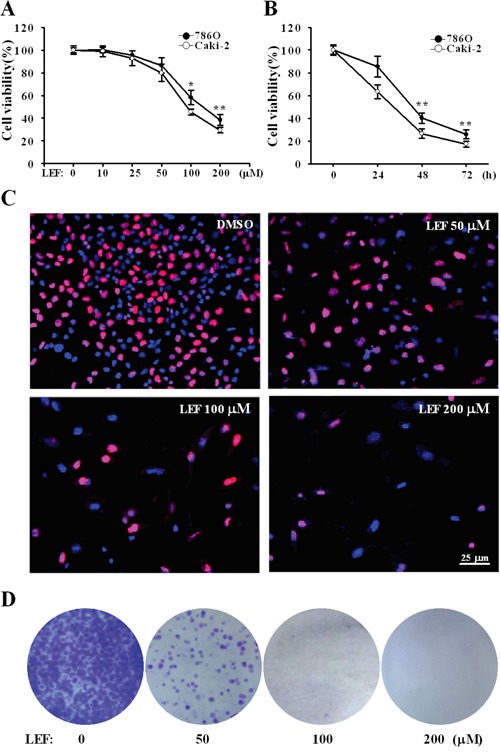
LEF reduces cell viability and cell growth in RCC cells **A.** Cell viability was estimated by MST assay after Caki-2 and 786O cells were incubated with increasing concentrations of LEF for 48 h. DMSO was used as a control. **B.** The time-response curve of 200 μM LEF on cell viability of Caki-2 and 786O cells. Data in A and B represent mean ± SD from three independent experiments (*P<0.01, ** *P*<0.05, vs. the control). **C.** EdU incorporation assay was analyzed by fluorescence microscopy in Caki-2 cells treated with elevated concentrations of LEF (0-200 μM) for 48 h. Nuclei were visualized with Hoechst 33342. **D.** Representative images of cell colony formation assay to evaluate the long-term growth inhibition effects of LEF. Caki-2 cells were maintained in indicated concentrations of LEF for 7 days before staining with crystal violet.

In order to evaluate the effects of LEF on RCC cell lines, cell viability was tested in Caki-2 and 786O cell lines by MTS assay. After exposure to elevated concentrations of LEF (0-200 μM) for 48 h, both of the tested RCC cell lines showed dose-dependent decrease in cell viability (Figure 1A). Comparatively, Caki-2 cells were more sensitive to LEF administration than 786O cells. It is well known that LEF at low concentrations (IC_50_ 1–3 μM) can block the enzymatic activity of DHODH, thereby inhibiting pyrimidine synthesis. However, our results suggested that LEF at 10 and 25 μM did not exert significant effect on cell viability. Compared with the DMSO-treated control, viability of Caki-2 cells was decreased to about 79.8% and 45.5% after treatment with 50 and 100 μM LEF for 48 h, respectively. Maximal decrease in cell viability to about 29.4% was achieved in Caki-2 cells after incubation with 200 μM LEF. MTS assays also revealed that exposure to 100 μM LEF resulted in significant dose-dependent reduction in cell viability (Figure 1B).

Next we employed the EdU incorporation assay to detect DNA synthesis. After treatment with increasing concentrations of LEF for 48 h, the number of EdU positive cells significantly decreased in a dose-dependent manner (Figure 1C). The statistical analysis revealed that the number of EdU positive cells in treatment group of LEF at 200 μM was reduced by 60% relative to that of the control cells (data not shown). These results indicate that LEF can inhibit the proliferation of Caki-2 cells. Colony formation assays further confirmed that long-time treatment (7 days) with LEF at concentrations exceeding 50 μM almost completely inhibited the expansion of tumor clones from a single cell (Figure 1D). LEF at low concentrations could cause a decrease in colony number. Likewise, LEF can greatly impede the growth of 786O cells (data not shown).

**Figure 2:**
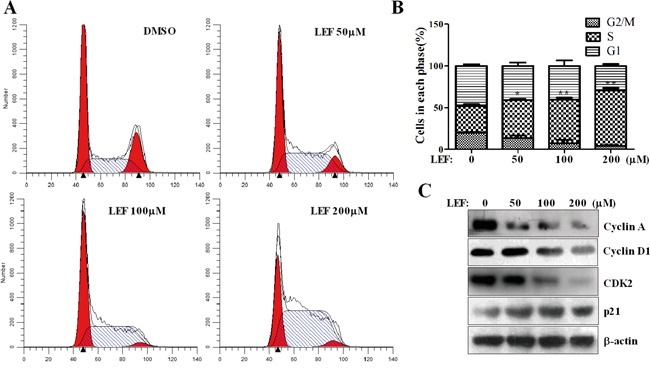
LEF induces cell-cycle arrest **A.** After LEF treatment for 48 h, Caki-2 cells were stained with PI and subjected to cell cycle analysis by flow cytometry. One representative experiment out of three is shown. **B.** The statistical analysis of cell percentage of cell-cycle distribution. Data represent mean ± SD of three independent experiments (*P<0.01, ** *P*<0.05, vs. the control). **C.** Changes in cell-cycle regulatory proteins after LEF treatment for 48 h. Representative images from at least three independent experiments are shown.

To further investigate the impact of LEF on the proliferation of Caki-2 cells, the cell cycle profile was analyzed using propidium iodide staining and flow cytometry after LEF treatment for 48 h. Compared with the control, LEF treatment caused a dose-associated accumulation in S-phase arrest accompanied by a decreased cell population in G2/M phases (Figure 2A and 2B). Data showed that the proportion of control cells in the S phase was 31.56±2.52%. This value reached 45.54±1.39%, 52.07±2.63%, and 66.18±3.09% in groups treated with 50, 100, and 200 μM LEF, respectively. Additionally, 200 μM LEF greatly decreased the cell population in the G2/M phase (Figure 2A and 2B). The cell proportion of G2/M phase declined from 20.03±0.65% in control group to 4.12±0.67% with 200 μM LEF. These data provide strong evidence that LEF inhibits proliferation of RCC cells by inducing S-phase cell cycle arrest. The functional association of cyclin A with CDK2 is required for cell cycle progression through the S phase. Accordingly, LEF treatment was accompanied by a decrease of cyclin A and CDK2 (Figure 2C). p21 protein, a CDK inhibitor, was up-regulated after LEF administration. In comparison, the high concentrations of LEF could exert the significant inhibition on the expression of cyclin D1, thereby aggravating the cell growth arrest. Seemingly, there are distinct characteristics among different concentrations of LEF with regard to growth inhibition.

### LEF induces cell apoptosis and autophagy

**Figure 3:**
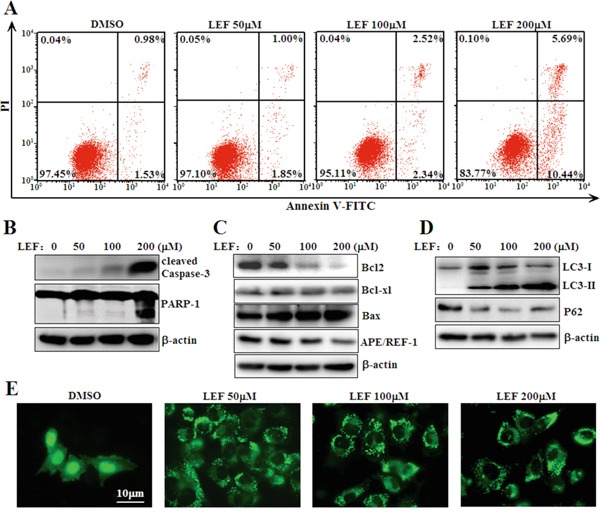
LEF triggers cell apoptosis and autophagy **A.** Flow cytometry analysis of apoptosis was determined using Annexin V-FITC/PI staining in Caki-2 cells treated with LEF for 48 h. Data are typical of three similar experiments. The percentage of Annexin V-FITC and/or PI positive cells was depicted with cytofluorometer quadrant graphs. **B.** LEF treatment induced cleavage of caspase-3 and PARP-1 as indicative of apoptosis. **C.** Expression alterations of anti-apoptotic and pro-apoptotic proteins after LEF treatment for 48 h. **D.** Changes in autophagy-associated proteins after LEF treatment for 48 h. Representative images in B, C, and D, are from at least three independent experiments. **E.** Visualization of GFP-LC3 fluorescence in Caki-2 cells after LEF treatment for 48 h.

Subsequently, we explored whether LEF could mediate apoptosis induction beyond growth inhibition. After incubation with increasing concentrations of LEF for 48 h, cells were stained with Annexin V-FITC and propidium iodide and analyzed by flow cytometry. Cells stained neither by Annexin V-FITC nor by PI were considered viable. As shown in Figure 3A, few apoptotic cells occurred after treatment with 50 and 100 μM LEF. Cell apoptosis was moderately induced in 200 μM LEF group. Next, immunoblotting assay was performed to investigate the expression of apoptosis related proteins. As expected, 200 μM LEF triggered the cleavage of PARP-1, a hallmark of apoptosis (Figure 3B). The amount of active Caspase-3, accounting for PARP cleavage, was elevated with increasing dose of LEF (Figure 3B). Coherent with data from flow cytometry, the most significant cleavage of Caspase-3 and PARP-1 was observed in 200 μM LEF group. Further, pro-apoptotic and anti-apoptotic proteins were examined by immunoblotting assay. As shown in Figure 3C, the expression of the anti-apoptotic Bcl2 and APE/REF-1 proteins was downregulated by LEF treatment at high concentrations. Conversely, the pro-apoptotic protein Bax was induced. Another anti-apoptotic protein Bcl-xl was nearly unaffected by LEF.

Moreover, we also observed that LEF could trigger autophagy in Caki-2 cells. Upon treatment with LEF, Caki-2 cells exhibited a marked elevation of LC3-II and a decrease of P62 in protein levels (Figure 3D). Meanwhile, Caki-2 cells transfected with LC3-GFP plasmids manifested a phenotypic relocalization of LC3-GFP after LEF treatment. In the absence of LEF, LC3-GFP expression was predominantly diffuse. LEF treatment resulted in the accumulation of LC3 puncta in the cytoplasm (Figure 3E). Unlike LEF-induced cell apoptosis, 50 μM LEF was sufficient to induce autophagy in Caki-2 cells.

### LEF inhibits WNT/β-catenin pathway

**Figure 4:**
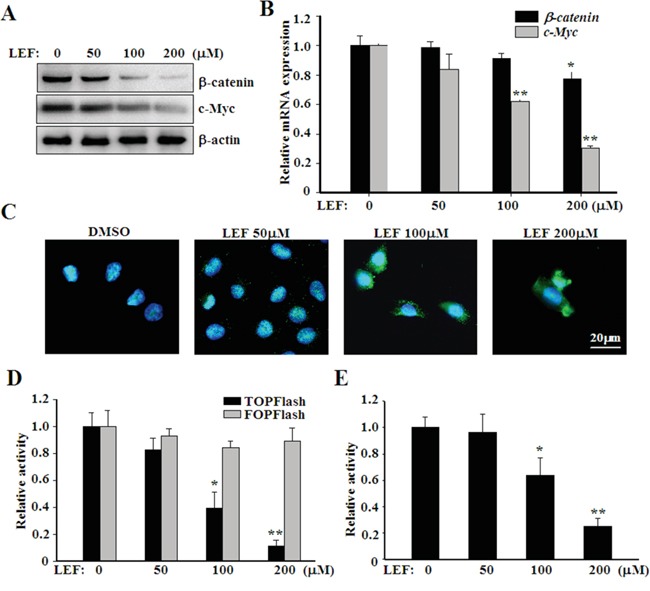
LEF inhibits canonical WNT/β-catenin signaling **A.** Western blot assay showing dose-dependent effect of LEF on β-catenin and c-Myc protein levels in Caki-2 cells. **B.** Real-time PCR for the expression of *β-catenin* and *c-Myc* mRNA levels. Data represent mean ± SD from three independent experiments. **C.** LEF induced the translocation of β-catenin from the nucleus into the cytoplasm in Caki-2 cells. **D.** Luciferase assay to estimate the activation of canonical WNT/β-catenin signaling. Caki-2 cells were transiently transfected with TOPFlash or FOPFlash constructs (1 μg), both in combination with pRSVluc plasmid as an internal control. 6 h after transfection, cells were subsequently treated with depicted concentrations of LEF for another 48 h. **E.** The transcriptional activity of *c-Myc* promoter was analyzed by luciferase reporter assay. Luciferase activity in D and E was measured and normalized to Renilla luciferase activity. All experiments were done in triplicates and each bar represents mean ± SD (*P<0.01, ** *P*<0.05, vs. the control).

Previous reports have highlighted that LEF can affect cell proliferation and survival via mechanisms other than DHODH inhibition. Given the significance of canonical WNT/β-catenin pathway in tumorigenesis, we next investigated the impact of LEF on the canonical WNT/β-catenin pathway. As shown in Figure 4A, high concentrations of LEF caused a remarkable decrease of β-catenin proteins. By contrast, LEF preferred to affect the protein abundance of β-catenin rather than its mRNA expression (Figure 4B). c-Myc, a known β-catenin downstream target, was significantly downregulated in mRNA and protein levels by LEF treatment. Furthermore, we observed that LEF could induce significant nuclear export of β-catenin, which is a feature of canonical WNT inhibition (Figure 4C). Importantly, β-catenin shuttled from the nucleus into the cytoplasm after LEF administration and exhibited a speckled cytoplasmic distribution, which may represent the formation of β-catenin destruction complex. Furthermore, TOPFlash and FOPFlash constructs were transiently transfected to evaluate β-catenin-dependent transcriptional activation in Caki-2 cells (Figure 4D). LEF treatment gradually abrogated the transcriptional activity of TOPFlash, but not FOPFlash constructs. Likewise, LEF treatment at high concentrations also reduced the luciferase activity of c-Myc reporter (Figure 4E). Together, these data indicate that LEF can inhibit the activation of WNT/β-catenin pathway in renal cell carcinoma.

### LEF induces β-catenin degradation via AKT inhibition

**Figure 5:**
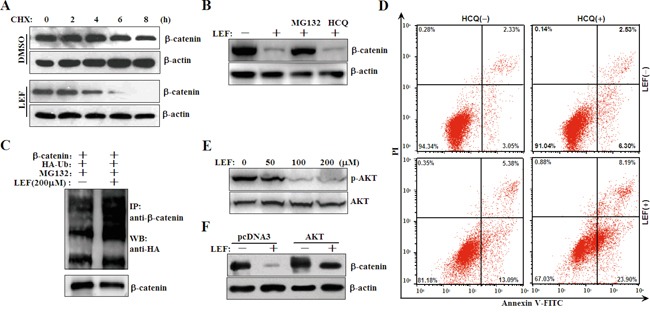
LEF represses AKT kinase to induce β-catenin degradation **A.** Accelerated degradation of β-catenin protein after LEF treatment in Caki-2 cells. Caki-2 cells were treated with 200 μM LEF for 48 h, and then incubated with 20 μM CHX for indicated periods. The cell extracts were subjected for Western blot to analyze the rate of β-catenin degradation. **B.** Caki-2 cells were treated with 200 μM LEF together with 10 μM MG132 or 10 μM HCQ for 48 h. **C.** Ubiquitylation of β-catenin by LEF. Caki-2 cells were transfected with β-catenin together with HA-tagged ubiquitin (HA-Ub) vectors. Immunoprecipitation was conducted with anti-β-catenin and subsequently immunoblotting analysis was performed with anti-HA antibodies. **D.** Flow cytometry analysis of apoptosis was determined in Caki-2 cells treated with 200 μM LEF and 10 μM HCQ for 48 h. Data are typical of three similar experiments. The percentage of Annexin V-FITC and/or PI positive cells was depicted with cytofluorometer quadrant graphs. **E.** Western blot detected the protein levels of phosphorylated and total AKT in Caki-2 cells treated with 200 μM LEF for 48 h. **F.** Caki-2 cells were transfected with AKT plasmids or the control constructs, and then cells were treated with 200 μM LEF for 48 h to detect the degradation of β-catenin with Western blot. Representative images from at least three independent experiments are shown.

Our results implicated that LEF might interrupt the protein stability and nucleo-cytoplasmic distribution of β-catenin rather than repress its expression. To verify this, Caki-2 cells were treated with 200 μM LEF for 24 h, and then subjected to a protein synthesis inhibitor, cycloheximide (CHX). Cell extracts were isolated at indicated time points and subjected to immunoblotting for the detection of β-catenin degradation. As shown in Figure 5A, the degradation of β-catenin was greatly accelerated upon LEF treatment. Given that both of ubiquitin-proteasome and autophagy-lysosome pathways are capable to eliminate β-catenin, we subsequently ascertained which one was responsible for LEF-induced β-catenin degradation [18]. As shown in Figure 5B, MG-132, an inhibitor of ubiquitin-proteasome system, but not autophagy inhibitor HCQ, significantly reversed LEF-induced β-catenin degradation. After LEF treatment, β-catenin was greatly polyubiquitylated (Figure 5C). Thus, our results showed that LEF facilitated the degradation of β-catenin protein via the ubiquitin-proteasome pathway. Nevertheless, the combination of HCQ and LEF enhanced LEF-induced cell death in Caki-2 cells (Figure 5D). Thus, although autophagy is irrelevant with LEF-induced β-catenin degradation, it plays a cytoprotective role to abrogate LEF-mediated apoptosis.

AKT and GSK3β exert a positive and negative effect on β-catenin stabilization and localization, respectively. In this study, we detected that LEF treatment at 100 and 200 μM effectively inhibited the phosphorylation of AKT kinase (Figure 5E). Conversely, overexpression of AKT1 plasmids partially ameliorated the LEF-mediated reduction in β-catenin (Figure 5F). Therefore, LEF-induced β-catenin degradation attributed to AKT inhibition.

### LEF upregulates WNT3a to recover WNT/β-catenin signaling

**Figure 6:**
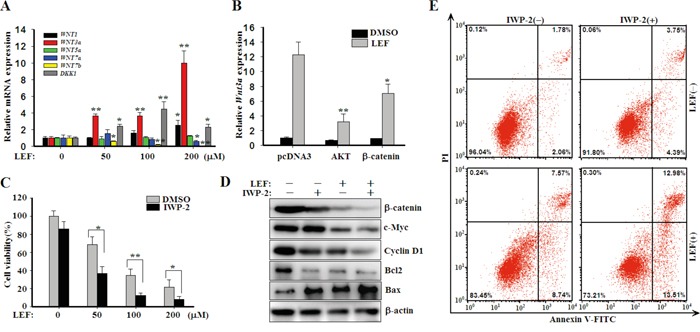
LEF upregulates WNT ligands to compromise cytotoxic effects **A.** Real-time PCR for the expression of *WNT1, WNT3a, WNT5a*, *WNT7a*, *WNT7b*, and *DKK1* in mRNA levels. Data represent mean ± SD from three independent experiments. **B.** Caki-2 cells were transfected with plasmids encoding AKT or β-catenin as depicted, and then cells were treated with 200 μM LEF for 48 h to detect the expression of *WNT3a* mRNA by real-time PCR. **C.** Cell viability was estimated by MST assay after Caki-2 acells were incubated with increasing concentrations of LEF together with 20 μM IWP-2 for 48 h. All experiments were done in triplicates and each bar represents mean ± SD (*P<0.01, ** *P*<0.05, vs. the control). **D.** Changes of growth and apoptosis-associated proteins after combined treatment of LEF and IWP-2 for 48 h. Representative images from at least three independent experiments are shown. **E.** Flow cytometry analysis of apoptosis was determined in Caki-2 cells treated with 200 μM LEF and 20 μM IWP-2 for 48 h. Data are typical of three similar experiments. The percentage of Annexin V-FITC and/or PI positive cells was depicted with cytofluorometer quadrant graphs.

Subsequently, we investigated whether LEF affects the expression of WNT ligands and antagonists to inhibit canonical WNT/β-catenin pathway. Intriguingly, LEF treatment greatly enhanced the expression of *WNT3a* and *DKK1* (Figure 6A). While the mRNA transcript of *WNT1* and *WNT5a* was slightly affected by LEF, and the mRNA levels of *WNT7a* and *WNT7b* decreased under LEF treatment. We further speculated that the LEF-mediated upregulation of *WNT3a* might be a negative feedback of AKT or β-catenin inhibition. After transfection with plasmids encoding AKT1 or β-catenin, Caki-2 cells were then incubated with 200 μM LEF for 48 h and mRNA was extracted for real-time PCR. As shown in Figure 6B, AKT1 or β-catenin overexpression impeded LEF-induced *WNT3a* upregulation.

Presumably, upregulated *WNT3a* can rescue the repressed activity of WNT/β-catenin pathway to promote cell proliferation and survival. Hence, we treated Caki-2 cells with LEF together with IWP-2, an inhibitor of WNT processing and secretion. As expected, IWP-2 significantly enhanced the anti-proliferative effect of LEF (Figure 6C). It was also obvious that the combination of LEF and IWP-2 could minimize the expression of β-catenin, c-Myc, Cyclin D1, Bcl2 and Bax to the largest extent compared with single agents (Figure 6D). Though IWP-2 almost unaffected cell apoptosis, the combination treatment had a greater pro-apoptotic effect in Caki-2 cells (Figure 6E). Taken together, our results revealed that LEF treatment can upregulate *WNT3a* expression to counteract the anti-proliferative and pro-apoptotic effects of LEF.

### LEF downregulates FZD10 expression

**Figure 7:**
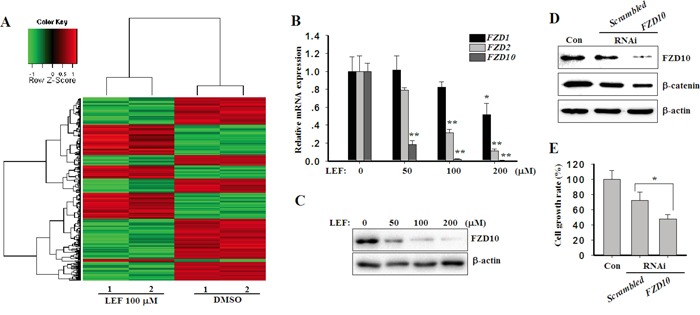
LEF decreases the expression of FZD10 **A.** Heatmap of hierarchical clustering of gene expression from Caki-2 cells treated with 200 μM LEF or vehicle control. **B.** Real-time PCR for the expression of *FZD1, FZD2*, and *FZD10* in mRNA levels after LEF treatment. Data represent mean ± SD from three independent experiments **C.** Changes of FZD10 proteins after LEF treatment for 48 h. **D.** Caki-2 cells were transfected with FZD10 or scrambled siRNAs respectively. After 48 h, western blot detected the protein levels of FZD10 and β-catenin. Representative images from at least three independent experiments are shown. **E.** Cell growth was estimated by MST assay after siRNA transfection for 72 h. (*P<0.01, ** *P*<0.05, vs. the control).

To further identify the pharmacological targets of LEF, we examined the transcriptional consequences of LEF treatment in Caki-2 cells utilizing gene expression microarray. As shown in Figure 7A, 175 genes were significantly downregulated after LEF treatment, whereas 114 genes were upregulated by more than 2-fold. Among them, FZD10, a WNT receptor, was found as a major target of LEF treatment. Its expression was dramatically decreased by more than 800-fold after LEF treatment. Real-time PCR further validated that *FZD10* expression was greatly repressed by LEF treatment (Figure 7B). In comparison, the mRNA levels of *FZD1* and *FZD2* were moderately reduced by LEF. Immunoblotting assay also confirmed that the inhibitory effect of LEF on FZD10 expression (Figure 7C). Subsequent experiments indicated that FZD10 can positively modulate cell proliferation via the activation of Wnt/β-catenin signaling. Specific siRNAs specifically downregulated the expression of *FZD10*, thereby leading to a decrease of β-catenin (Figure 7D). RNAi targeting *FZD10* also caused an inhibition in cell growth (Figure 7E). Taken together, our results suggest that LEF treatment suppresses the expression of WNT receptors and ligands, such as *WNT7a*, *WNT7b, FZD1*, *FZD2*, and *FZD10*, and augments *DKK1* expression. This mechanism may account for the inhibition of canonical WNT/β-catenin pathway.

### LEF inhibits tumor growth in mouse xenograft model

**Figure 8:**
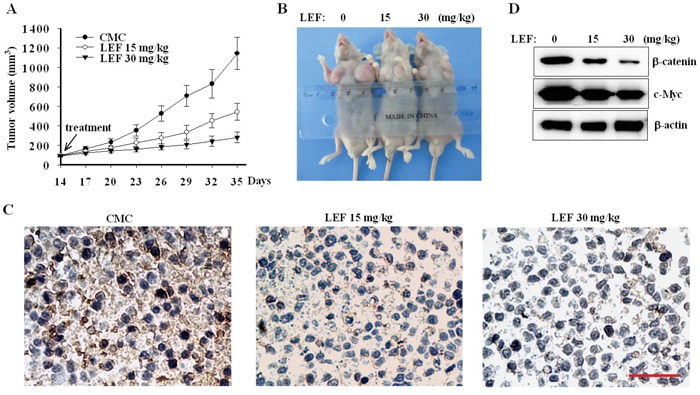
LEF suppresses xenograft tumors in mice model **A.** The rates of xenograft growth were monitored at indicated days in NOD/SCID mice receiving LEF or vehicle. Error bars represent standard deviations, n = 8. **B.** Tumor samples showing final tumor size after mice were sacrificed. **C.** Representative immunohistochemical staining of FZD10 in xenograft tissues. Scale bar 50 μm. **D.** Immunoblotting analysis of β-catenin and c-Myc from xenograft tumors treated with LEF or vehicle (n = 5). One representative experiment out of three is shown.

To evaluate anti-tumor activity of LEF *in vivo*, Caki-2 cells were injected into immunodeficiency NOD/SCID mice. 2 weeks after tumor implantation, NOD/SCID mice developed rapidly growing tumors, and then orally received 2% CMC solution or 15, 30 mg/kg LEF daily for another 3 weeks. As seen in Figure 8A and 8B, the rates of tumor growth dramatically declined in LEF groups compared with the control mice. LEF administration (15 and 30 mg/kg) led to about 52 and 75% decrease in tumor size compared with the control groups, respectively. Moreover, the mice body weight was not affected by LEF administration, implying that LEF did not exert any severe side effect on mice (data not shown). As expected, LEF effectively reduced the expression of FZD10 as demonstrated by immunohistochemical staining (Figure 8C). Immunoblotting assay also confirmed that LEF decreased the expression of β-catenin and c-Myc (Figure 8D). Therefore, our current results validate anti-tumor effects of LEF in mice transplanted with RCC cells, and reveal that β-catenin inhibition may be an important mechanism of LEF-mediated suppression on xenografts.

## DISCUSSION

LEF is an eminent agent in RA and transplantation medicine owing to its combined immunosuppression, anti-inflammatory, and anti-viral benefits. Emerging evidence unequivocally identifies its potential to antagonize tumorigenesis and induce apoptosis. However, the modes of LEF action responsible for its anti-tumor effects remain controversial. In this study, we demonstrated that LEF exerts its cytotoxicity on RCC cells through inducing growth arrest, autophagy and apoptosis at increasing concentrations. Canonical WNT/β-catenin pathway was characterized as a pharmacological target of LEF at high concentrations.

It is well known that the mitochondrial enzyme DHODH is a major target of LEF and its active metabolite, teriflunomide. DHODH is required for de novo pyrimidine synthesis, which is indispensable in proliferating cells to meet their augmented demand for nucleic acid precursors. This mechanism also plays a crucial role in aberrant proliferation of malignant cells. The inhibition of DHODH results in growth arrest of proliferating cells at the G0/G1 or G1/S phase cell-cycle transition. At low concentrations (IC_50_ 1–3 μM), LEF reversibly binds to DHODH and causes its repression. Exogenous uridine can reverse the anti-proliferative effects at these concentrations. However, at high concentrations of LEF or A771726 (>50 μM), uridine administration no longer completely reverses the anti-proliferative effects, implicating that there are other mechanisms contributing to LEF-mediated growth inhibition [2, 19-21].

Indeed, other targets have been identified and may account for the anti-proliferative effects of LEF regardless of the availability of pyrimidines. Another putative mode of LEF action could be the inhibition of kinases involved in cell proliferation and activation. Tyrosine kinases (e.g., PDGFR, EGFR, and JAK), serine/threonine kinases (e.g., AKT, p70S6K1, and PDK1), and phospholipases (e.g., PLCγ1) can be inhibited by LEF and A771726, thereby interfering with cellular behavior and responses to exogenetic signals [2, 3, 5, 22-25]. LFM-A13, a LEF metabolite analog, has documented as a selective inhibitor of polo-like kinase (PLK) [6]. It is well characterized that the dose for LEF and its analogs to inhibit kinases is about 50-150 μM, approximately 10- to 100-fold higher than that required to abrogate pyrimidine synthesis. Meanwhile, LEF can effectively inhibit the activation of NF-κB by blocking degradation of IkBα, contributing to the downregulation of inflammatory or proliferative cytokines such as TNF-α and IL-6 [26, 27].

In this study, we observed that LEF exerted different effects on cell proliferation and survival in RCC cells at varying concentrations. At 50 μM, LEF can effectively induce S phase arrest to achieve growth inhibition. Simultaneously, LEF triggers cell autophagy, thereby antagonizing cell apoptosis. By comparison, 200 μM LEF induces S phase arrest and further represses G2/M phase progression, and significantly elicits cell apoptosis. Obviously, these data indicate that high concentrations of LEF can further aggravate cell viability via other mechanisms independently of DHODH inhibition. Indeed, our results reveal that only high concentrations of LEF strongly affect the expression of several genes associated with cell growth and survival, such as Cyclin D1, CDK2, c-Myc, Bcl2, and APE/REF-1. Comparatively, the expression of p21, Cyclin A, p62, and LC3-II is readily altered by 50 μM LEF. Further studies strongly suggest that LEF treatment at high concentrations render an abrogation of the canonical WNT/β-catenin pathway. In fact, LEF inhibited β-catenin expression in *in vitro* cultured cells and tumor tissues derived from NOD/SCID null mice. The inhibition of WNT/β-catenin pathway might be a putative mechanistic rationale for LEF on cell growth arrest and apoptosis induction.

WNT signaling is extensively involved in various cell processes such as proliferation, migration, differentiation, motility, and survival. WNT proteins are a conserved family of secreted, extensively palmitoylated glycoproteins. WNT ligands function through binding with a heterodimeric receptor complex comprised of seven-transmembrane Frizzled (FZD) receptors and the coreceptors low-density LRP5/6. Until now, 19 WNT ligands and 10 FZD receptors have been unearthed in mammals. WNT signaling can be categorized into the β-catenin dependent (canonical), and β-catenin independent (non-canonical) pathways. β-catenin, the key downstream regulator of the WNT pathway, is activated when any of the canonical pathway ligands bind to FZD and the LRP5/6 complex, thereby preventing its phosphorylation-dependent degradation. Ultimately, it serves as a coactivator of the transcription factors TCF/LEF to promote the transcription of target genes governing cell proliferation such as *Cyclin D1* and *c-Myc*. β-catenin phosphorylated by glycogen synthase kinase (GSK3β), is then ubiquitinated by an E3 ubiquitin ligase, β-TrCP, and degraded by the proteasome.

Multiple studies have shown that aberrant activation of WNT signaling pathway is a major etiological factor in multiple tumor types including RCC. WNTs and FZDs have been found to be constitutively active in RCC, implicating an autocrine and/or paracrine pathway for aggressive growth and invasion [28, 29]. In clear cell RCCs, upregulated WNT1/β-catenin signaling was associated with unfavorable clinicopathology and impaired survival [30]. Moreover, the APC gene promoter is hypermethylated in a subset of RCCs, which means β-catenin is dissociated from the destruction complex and activates its transcriptional function via translocating into the nucleus [31]. The mutation or loss of von Hippel–Lindau (VHL) tumor suppressor gene also activates β-catenin via the HGF-PI3K-AKT cascade [32]. Several classes of secreted proteins exist as natural antagonists for WNT signaling, such as Dickkopf-related proteins (DKKs) and secreted Frizzled-related proteins (sFRPs). However, the promoters of several endogenous WNT antagonists are epigenetically silenced in primary RCC samples when compared to the corresponding normal renal tissue samples [33]. These alterations converge into abnormal activation of WNT signaling in RCCs, promoting tumorigenicity, proliferative rate, metastatic potential, and insensitivity to radio-chemotherapy.

Herein, our results showed that LEF at high concentrations targets the canonical WNT/β-catenin signaling. LEF treatment induced the nucleo-cytoplasmic shuttling of β-catenin and subsequently promoted its proteasome-dependent proteolysis. Thereby, LEF can impair the transcriptional network of β-catenin, accounting for the decrease of c-Myc and Cyclin D1. Mechanistic studies revealed that high concentrations of LEF inhibited the phosphorylation of AKT kinase, which can oppose GSK3β to maintain β-catenin activity. This finding is consistent with previous reports that LEF can affect tyrosine and serine/threonine kinases including AKT. Furthermore, our studies uncovered that LEF treatment can evoke extensive changes of WNT ligands and receptors in mRNA levels. LEF treatment can inhibit the expression of *WNT7a*, *WNT7b*, *FZD2*, and *FZD10*. At the same time, LEF induced the upregulation of *WNT3a* and *DKK1*. Importantly, FZD10 was identified as one of the most repressed genes by LEF treatment. Previous reports suggested that FZD10 mediates WNT1, WNT3a, WNT7a, WNT7b, and hypoxia-inducible protein-2 signaling in embryonic development and other biological events [34-37]. However, FZD10 is low or absent in vital organs including the brain, heart, lung, liver, kidney, and bone marrow. Conversely, upregulation of FZD10 has been reported in multiple tumors such as colorectal cancer, gastric cancer, cervical cancer, breast cancer, and synovial sarcoma, whereas the precise outcome of FZD10 in RCC largely remains obscure. Tumor growth could be attenuated by targeting FZD10 through small-interfering RNA or humanized antibodies or by inducing epigenetic silence of FZD10 [38-40]. Because FZD10 expression is rare in vital organs, adverse reactions would be minimized. In addition to decreasing FZD10, we observed LEF-mediated repression of *FZD1* and *FZD2*. Taken together, the inhibitory effects of LEF on Frizzled receptors may be a conceivable mechanism to block WNT/β-catenin signaling.

Instead, the mRNA level of *WNT3a* gene was significantly increased after LEF treatment. The inducible expression of WNT3a was partially derived from AKT or β-catenin inhibition, thereby weakening its negative feedback regulation. Aberrant hyperactivation of WNT3a has been shown to be closely associated with tumor progression and clinical grade in various cancer types, however its mechanism of action varies significantly depending upon tumor type [41]. In multiple tumor types, WNT3a is capable of promoting the proliferation of tumor cells via canonical WNT/β-catenin signaling. Additionally, WNT3a antagonized the growth inhibition of liver cancer stem cells induced by 8-bromo-7-methoxychrysin [42]. WNT3a was able to reverse docosahexaenoic acid-induced growth inhibition in human pancreatic cancer PANC-1 cells [43]. In 4T1 murine mammary cancer cells, WNT3a was found to restore the suppressed cell viability by quercetin [44]. Moreover, WNT3a treatment significantly reduced the sensibility of cholangiocarcinoma QBC939 cells to chemotherapeutics [45]. Therefore, WNT3a seems to be an important secreted signaling molecule conferring resistance to cytotoxic agents. In this study, the combined treatment of LEF and IWP-2 can further reduce the viability of Caki-2 cells and induce cell apoptosis. This result may highlight a feasible approach to potentiate the therapeutic effects of LEF.

Overall, our findings indicate that LEF can inhibit the viability of RCC cells. High concentrations of LEF can interrupt the canonical WNT/β-catenin signaling to induce growth arrest and apoptosis. Thus, LEF could serve as a therapeutic agent for RCC.

## MATERIALS AND METHODS

### Reagents and plasmids

Leflunonmide (LEF) was obtained from Sigma-Aldrich. Hydroxychloroquine (HCQ) and IWP-2 were purchased from J&K chemical Ltd and Selleck Chemicals, respectively. Antibodies against microtubule-associated protein 1 light chain 3 (LC-3), P62, Caspase-3, PARP-1, Bcl2, Bcl-xl, Bax, phospho-AKT, and total AKT were obtained from Cell Signaling Technology. Antibodies specific for HA, Cyclin A, CyclinD1, CDK2, p21, APE/REF-1, c-Myc, β-catenin, FZD10, and β-actin, were purchased from Santa Cruz Biotechnology. The luciferase reporter constructs of c-Myc-luc, TOPFlash, and FOPFlash have been described in previous reports [17]. The plasmid encoding β-catenin, AKT1, LC3-GFP, and HA-Ub were kindly provided by Dr. Chenguang Zhang (College of Basic Medicine, Tianjin Capital Medical University) or from from Addgene (Boston, MA).

### Cell culture

The human RCC cell lines 786O and Caki-2 were purchased from the Shanghai Institute of Cell Biology (Shanghai, China) and were cultured in RPMI 1640 medium supplemented with 10% fetal bovine serum (Gibco), penicillin (100 U/ml), and streptomycin (100 μg/ml) under a humidified atmosphere containing 5% CO2 maintained at 37°C.

### MTS assay and colony formation assay

Cell viability was determined by MTS assay. Cells (5×10^3^) were seeded into 96-well plates and treated with LEF or other agents for depicted time intervals. After treatment, 10 μl MTS (Promega, USA) was added into each well for 2 h incubation. The absorbance was measured using a model ELX800 Micro Plate Reader (Bio-Tek Instruments, USA) at 490 nm. For colony formation assay, Caki-2 cells were trypsinized to single cell suspensions and seeded into fresh 6-well plates at 1000 cells/well. Then cells were incubated with LEF at depicted concentrations for 7 days. Colonies were fixed with absolute methanol for 15 min and then stained with 0.1% crystal violet for 20 min. After washing with PBS three times, the colonies with a diameter over 2 mm were visualized by a digital camera (Canon, USA).

### EdU incorporation assay

Cell proliferation was determined by incorporation of 5-ethynyl-2′-deoxyuridine (EdU) using an EdU Cell Proliferation Assay Kit (Ribobio, China). Cells were cultured in triplicate in 24-well plates at a density of 5×10^4^ and treated with LEF for 48 h at 37°C, and then 50 mM EdU was added to each well and cells were cultured for additional 2 h at 37°C. The cells were fixed with 4% formaldehyde for 30 min at room temperature and treated with 2 mg/ml glycine for 5 min. After washing with PBS for 5 min, the cells were treated with 0.5% Triton X-100 for 10 min at room temperature for permeabilization. 100 μl of 1X Apollo^®^ reaction cocktail was added to each well and the cells were incubated for 30 min in the dark at room temperature. After washing with Triton X-100 three times, the cells were washed by methanol and PBS. Then the cells were stained with 100 μl Hoechst33342 for 30 min in the dark and then washed with PBS. Visualize under a fluorescent microscope (Olympus Corporation, Japan). The EdU positive cells (red cells) were counted using Image-Pro Plus (IPP) 6.0 software (Media Cybernetics, USA). The EdU incorporation rate was expressed as the ratio of EdU positive cells to total Hoechst33342 positive cells (blue cells). All experiments were done in triplicate and three independent repeating experiments were performed.

### Flow cytometry analysis

To assess cell cycle progression by flow cytometry, Caki-2 cells (2×10^5^) after LEF treatment were suspended in 100 μl of PBS, and 200 μl of 95% ethanol were added while vortexing. The cells were then incubated at 4°C for 1 h, washed with PBS, resuspended in 250 μl of 1.12% sodium citrate buffer (pH 8.4) together with 12.5 μg of RNase, and incubated at 37°C for an additional 30 min. The cellular DNA was then stained by 250 μl propidium iodide (PI) for 30 min at room temperature. Red fluorescence emitted from the PI–DNA complex was analyzed at 488 nm/600 nm (excitation/emission wave length) using a FACScan flow cytometer (BD LSR II). The data of relative DNA content was analyzed by ModFit LT software package to ravel cell cycle distribution.

### Apoptosis assay

Caki-2 cells (2×10^5^) were seeded in 6-well plates and allowed to attach overnight. After LEF treatment in the presence or absence of other agents, cells were harvested and resuspended in 200 μl binding buffer after washing twice with cold PBS. Then, apoptotic cells were measured by an Annexin-V apoptosis detection kit (MultiSciences Biotech, China) according to the manufacturer's protocol. The cell suspension was incubated with 10 μl Annexin V-FITC stock solution for 30 min at 4°C in the dark, then incubated with 5 μl PI solution for 5 min. Cell samples were analyzed by flow cytometry (BD LSR II) and apoptotic cell fractions were determined.

### Real-time PCR

Total RNA was isolated using the RNAiso Plus (Takara, China), and cDNA was prepared using Transcriptor First Strand cDNA Synthesis Kit (Roche, USA) according to the manufacturer's instructions. Quantitative reverse transcriptase PCR has performed to quantify the expression of interest genes using LightCycler^®^480 SYBR Green I Master (Roche, USA) according to the manufacturer's protocol on Roche light cycler version 3.5 (Roche, USA). PCR amplification has conducted with the following conditions: 1 cycle pre-incubation at 95°C for 10 min, followed by 45 cycles: denaturation for 10 sec at 95°C, annealing for 15 sec at 60°C, and extension for 15 sec at 72°C. The primer sequences are summarized in Table 1. The level of expression of each target gene was calculated using 2^-ΔΔCt^ method. The relative amount of each mRNA has normalized to *GAPDH*. Each sample has been examined in triplicate.

**Table 1:** Primer sequences used for Real-time PCR

Gene Name	Primer Sequences (5′→3′)
*β-catenin*	F: GCTTGTTCGTGCACATCAGGA R: TGTGAACATCCCGAGCTAGGA
*c-Myc*	F: GGTGCTCCATGAGGAGACAC R: GCAGAAGGTGATCCAGACTC
*WNT1*	F: GAACTGTCCCACTGCTCCAG R: GGATTCGATGGAACCTTCTG
*WNT3a*	F: ACTACGTGGAGATCATGCCC R: ATGAGCGTGTCACTGCAAAG
*WNT5a*	F: TGAATAACCCTGTTCAGATGTCA R: TGTACTGCATGTGGTCCTGA
*WNT7a*	F: CTGTGGCTGCGACAAAGAGAA R: GCCGTGGCACTTACATTCC
*WNT7b*	F: GAAGCAGGGCTACTACAACCA R: CGGCCTCATTGTTATGCAGGT
*DKK1*	F: CCTTGAACTCGGTTCTCAATTCC R: CAATGGTCTGGTACTTATTCCCG
*FZD1*	F: GGGGCTTAACAACGTGGAC R: CAGAAAGGACGTGCCGATAAA
*FZD2*	F: GTGCCATCCTATCTCAGCTACA R: CTGCATGTCTACCAAGTACGTG
*FZD10*	F: GGCGGTGAAGACCATCCTG R: CAGCTTGTCCGTGTTCTCG
*GAPDH*	F: CTCACCGGATGCACCAATGTT R: CGCGTTGCTCACAATGTTCAT

### Western blotting assay and immunoprecipitation

The cells (1 × 10^6^) were washed with cold PBS and lysed on ice in 100 μl modified RIPA buffer (50 mM Tris-HCl, pH 7.4, 1% NP-40, 0.25% Na-deoxycholate, 150 mM NaCl, 1 mM Na_3_VO_4_, and 1 mM NaF) containing protease inhibitors (100 μM phenylmethylsulfonyl fluoride, 10 μM leupeptin, 10 μM pepstatin, and 2 mM EDTA). The extracts were centrifuged at 12,000 × g for 10 min at 4°C, and the supernatant fractions were collected. For immunoprecipitation, cell lysates were precleared with Protein G-Sepharose (Roche, USA), and then immunoprecipitated with anti-β-catenin absorbed to protein G-Sepharose. The protein content in the supernatant was measured using a BCA Protein Assay Kit (Beyotime, China). The proteins were separated by SDS-PAGE electrophoresis and transferred to PVDF membranes (Millipore Corporation, USA), then blotted with specific secondary antibodies. Detection of specific proteins was carried out with an ECL chemiluminescence detection kit (Vigorous, China) according to the manufacturer's instruction. The figures shown are representative of at least three independent experiments.

### Immunofluorescence assay

Caki-2 cells were grown on coverslips and treated with either DMSO or LEF for 48 h. Then cell were fixed with 4% paraformaldehyde for 15 min at room temperature. After rinsing with PBS three times, cells were permeabilized with 0.3% Triton-X 100 (Sigma) for 15 min and then blocked with normal rabbit immune serum for 30 min at 37°C. The permeabilized Caki-2 cells were incubated with primary rabbit polyclonal antibody against β-catenin (Santa Cruz, USA, 1:200) overnight at 4°C and were then incubated with FITC-conjugated secondary antibody (Santa Cruz, USA, 1:200) for 30 min at room temperature. Nuclei were counterstained with Hoechst 33342 (Sigma) for 5 min at room temperature. Cells were examined using a fluorescent microscope (Olympus, Japan).

### Cell transfection, luciferase assay and RNA interference

Caki-2 cells were seeded into 24-well plates 24 h before transfection. Constructs noted in figure legends were transfected using Lipofectamine2000 (Invitrogen) according to the manufacturer's instruction. The total amount of DNA was kept constant using pcDNA3 plasmid. The pRSVluc plasmid (Promega) was cotransfected as an internal control. Luciferase activity was measured and normalized to Renilla luciferase activity. RNA interference-mediated silencing (RNAi) was performed using Lipofectamine RNAiMAX in OptiMEM (Invitrogen) according to the manufacturer's instructions. The FZD10 and scrambled siRNAs were purchased from RiboBio (RiboBio, China) and transfected at 100 nM. 6 hours after transfection, the medium was changed and cells were further cultured for 48 or 72 h for immunoblot or MTS assays. All experiments were done in triplicates and performed at least three times.

### Gene expression microarray

Caki-2 cells treated with 200 μM LEF or vehicle control for 48 h. Total mRNA from two independent sample of each group was extracted for gene expression analysis. Gene Expression Microarray was detected using RiboArrayTM genDETECTTM Human Array1×40K (Ribobio, China) according to the manufacturer's procedure. the dataset has been deposited in Gene Expression Omnibus (GEO), and accession numbers is GSE77433. To determine differentially expressed genes, gene expression levels were log2 transformed and then analyzed according to the manufacturer's protocol.

### *In vivo* tumorigenic assay and immunohistochemistry

This animal experiment was approved by the Institutional Animal Care and Use Committee at Zhejiang University. NOD/SCID mice were randomly allocated into 3 groups; 8 mice each. Caki-2 cells were harvested, suspended in PBS, and subcutaneously inoculated into the bilateral flanks of NOD/SCID mice (3×10^6^ cells per flank). After 2 weeks of tumor growth, the mice were intragastrically administered with LEF (15, 30 mg/kg/d) daily or 2% carboxy methyl cellulose (CMC) solution (5 ml/kg/day) as control for 21 days. Tumor diameter were measured in two dimensions with a caliper every three days, and tumor volume (mm^3^) was calculated as 0.4 × (short length)^2^ × long length. After treatment, mice were humanely sacrificed by cervical dislocation under anesthesia. One portion from each tumor was subjected for immunoblot analysis. Another portion from each tumor was fixed in 4% paraformaldehyd for 24 h at room temperature and then embedded into paraffin. Paraffin-embedded tissues were sectioned for immunohistochemistry (IHC) analysis using a 3, 3′-diaminobenzidine (DAB) detection kit (Zhongshan Biotech, China) in accordance with the manufacturer's protocol with anti-FZD10 antibody. Slides were photographed under a light microscope and the obtained images were directed for blind examination.

### Statistic analysis

All data in this study were displayed as means ± SD. Comparisons were analyzed by Student's *t*-test or one way ANOVA. The significance was analyzed with SPSS10.0 software and a *P*-value <0.05 was considered to be statistically significant.
